# SERS-PLSR Analysis of Vaginal Microflora: Towards the Spectral Library of Microorganisms

**DOI:** 10.3390/ijms232012576

**Published:** 2022-10-20

**Authors:** Sylwia Magdalena Berus, Monika Adamczyk-Popławska, Katarzyna Goździk, Grażyna Przedpełska, Tomasz R. Szymborski, Yuriy Stepanenko, Agnieszka Kamińska

**Affiliations:** 1 Institute of Physical Chemistry, Polish Academy of Sciences, Kasprzaka 44/52, 01-224 Warsaw, Poland; 2 Department of Molecular Virology, Faculty of Biology, University of Warsaw, Miecznikowa 1, 02-096 Warsaw, Poland; 3 Department of Parasitology, Faculty of Biology, University of Warsaw, Miecznikowa 1, 02-096 Warsaw, Poland; 4 Department of Dermatology and Venerology, Infant Jesus Clinical Hospital, Koszykowa 82a, 02-008 Warsaw, Poland

**Keywords:** surface-enhanced Raman spectroscopy (SERS), partial least square regression (PLSR), spectral library, vaginal microflora, *Lactobacillus* spp., *Bifidobacterium* spp., *Candida* spp., *Gardnerella vaginalis*, *Prevotella bivia*, *Trichomonas vaginalis*

## Abstract

The accurate identification of microorganisms belonging to vaginal microflora is crucial for establishing which microorganisms are responsible for microbial shifting from beneficial symbiotic to pathogenic bacteria and understanding pathogenesis leading to vaginosis and vaginal infections. In this study, we involved the surface-enhanced Raman spectroscopy (SERS) technique to compile the spectral signatures of the most significant microorganisms being part of the natural vaginal microbiota and some vaginal pathogens. Obtained data will supply our still developing spectral SERS database of microorganisms. The SERS results were assisted by Partial Least Squares Regression (PLSR), which visually discloses some dependencies between spectral images and hence their biochemical compositions of the outer structure. In our work, we focused on the most common and typical of the reproductive system microorganisms (*Lactobacillus* spp. and *Bifidobacterium* spp.) and vaginal pathogens: bacteria (e.g., *Gardnerella vaginalis*, *Prevotella bivia*, *Atopobium vaginae*), fungi (e.g., *Candida albicans*, *Candida glabrata*), and protozoa (*Trichomonas vaginalis*). The obtained results proved that each microorganism has its unique spectral fingerprint that differentiates it from the rest. Moreover, the discrimination was obtained at a high level of explained information by subsequent factors, e.g., in the inter-species distinction of *Candida* spp. the first three factors explain 98% of the variance in block Y with 95% of data within the X matrix, while in differentiation between *Lactobacillus* spp. and *Bifidobacterium* spp. (natural flora) and pathogen (e.g., *Candida glabrata*) the information is explained at the level of 45% of the Y matrix with 94% of original data. PLSR gave us insight into discriminating variables based on which the marker bands representing specific compounds in the outer structure of microorganisms were found: for *Lactobacillus* spp. 1400 cm^−1^, for fungi 905 and 1209 cm^−1^, and for protozoa 805, 890, 1062, 1185, 1300, 1555, and 1610 cm^−1^. Then, they can be used as significant marker bands in the analysis of clinical subjects, e.g., vaginal swabs.

## 1. Introduction

The vagina of a healthy woman is mainly inhabited by the facultative, microaerophilic or anaerobic *Lactobacillus* spp., which are essential in maintaining the proper state of the vagina and in producing metabolites such as acidolin, lactacin B, lactocidin, and hydrogen peroxide (H_2_O_2_) to prevent infections [1,2]. Verstraelen et al. related the stability of normal vaginal flora with the different species of lactobacilli. He documented that *Lactobacillus crispatus* as a H_2_O_2_-producer helps to maintain stable microflora, while *Lactobacillus gasseri* and *Lactobacillus iners* as non-H_2_O_2_ producers strongly favor the overgrowth of other bacteria, naturally leading to infections [3]. These results occurred to highly corroborate with those of Hawes et al., who claimed that only the decrease in the quantity of H_2_O_2_-producing lactobacilli leads to the acquisition and development of infections [4]. In some cases, a vagina can be primarily inhabited by *Bifidobacterium* spp. [5]. Such *Bifidobacterium*-dominated profiles are considered normal for reproductive-aged women and may have beneficial effects during birth, as these bacteria support infant health and development [6,7]. Although the presence of *Bifidobacterium* spp. in a human vagina is still an unresolved aspect, it is known that some of them have the ability to produce H_2_O_2_ that would maintain homeostasis (similarly to lactobacilli) [8].

Despite the abovementioned antimicrobial properties of natural flora (lactobacilli and bifidobacteria), the vagina may become dominated by fungi, anaerobic bacteria, and to a lesser extent by streptococci group A and B [9]. Such a change in the quantity and quality of inhabited microorganisms is known as vaginal infection. When the etiological dysbiosis causing agents are overgrowing bacteria (BV-related pathogens), e.g., *Gardnerella vaginalis*, *Prevotella* spp., *Atopobium vaginae*, *Mobiluncus* spp., it is defined as bacterial vaginosis (BV), while the fungal proliferation mainly of *Candida* spp. (*Candida albicans*, *Candida krusei*, *Candida glabrata*) may lead to vulvovaginal candidiasis (VVC). Both BV and VVC cause pain, itching, and abnormal vaginal discharge. On the contrary, Trichomoniasis (caused by motile protozoan *Trichomonas vaginalis*) is asymptomatic in one-third of cases, and because of the cytopathic effect, the mechanical barrier is breached, facilitating HIV transmission [10]. *Streptococcus agalactiae* is a group B streptococcus (GBS) known for its opportunistic properties that may cause neonatal sepsis and meningitidis if the woman’s vagina, cervix, or rectum is colonized by them [11,12].

The implementation of appropriate medicines strictly depends on the accurate differential diagnosis and is the only guarantor of effective treatment. Dysfunctions in the homeostasis of the genitourinary system may also predispose the development of sexually transmitted diseases (STDs) such as gonorrhea or chlamydiosis [13,14,15]. For all these reasons, determining the composition of vaginal flora and understanding its changes is a paramount aspect and has been of interest to many scientists so far.

Based on the grading of vaginal samples according to the Ison and Hay criteria (where grade: I—normal flora; II—intermediate flora; III—bacterial vaginosis (BV); IV—epithelial cells covered with Gram positive cocci) [16], Ellen de Becker et al. demonstrated that *L. crispatus* is present in all grades and mainly in grade I. Grade III is characterized by the highest concentration of *G. vaginalis* and *A. vaginae*. Since the presence of *A. vaginae* in other groups is rare, it seems to be a better indicator of bacterial vaginosis than *G. vaginalis*. Research has shown that the occurrence of *L. iners* promotes the growth of *G. vaginalis* (as there is a positive correlation between their concentrations). It is followed by the negative correlation between *L. gasseri* and *L. iners* [17]. As was proven, the mentioned species are synergistically related and extremely important in assessing vaginal microbiota. Therefore, in this article, our attention is mainly focused on the microorganisms mentioned so far.

There are some methods that allow the identification of bacteria. Gas–liquid chromatography (GLC) involves biochemical compounds present in bacteria cells or metabolites which are the basis of their discrimination. The most common compounds are: DNA, carbohydrates, membrane lipopolysaccharides, bacterial fatty acids, and proteins [18]. The main advantage of this method is the speed of analysis and the possibility of identification of bacteria that are no longer viable. This method was found to be accurate and was efficiently applied for the identification of *Lactobacillus* species without further verification [19]. The final results are obtained by comparison of many recordings, which implies that automated data processing is required. GLC involves a several-step procedure and chemical reagents which limits the technique. Some knowledge about Gram staining and bacteria morphology is needed.

Molecular techniques which utilize polymerase chain reaction (PCR) and amplification of mainly 16S rDNA are also introduced to microbiological diagnosis. In general, PCR itself is specific, but its shortcomings can interrupt the analysis. The primer selected at the start of the reaction detects only a particular group of microorganisms. Errors in amplifications can take place in the form of, e.g., binding of two primers redound to detection of nonspecific products. These organisms that have undergone genetic modification may yield misleading information (false-positive, false-negative results) [20,21]. Some modifications have been implemented to this technique that make it more accurate and reliable. Real-time PCR utilizes an optical module that enables the detection of the fluorescent product (amplicon) within the entire time of the reaction [21]. tDNA intergenic separation PCR (tDNA-PCR) uses primers that are complementary to the conserved edges of the tRNA genes and can amplify the intergenic spacers [22]. This technique was used to identify microorganisms forming vaginal microbiota [3,5] and lactobacillus species [22].

Raman spectroscopy is a technique that involves the effect of inelastic scattering of incident light by analyzed substances. As only one photon per million undergoes inelastic scattering, the Raman effect is very weak. Fleischmann’s studies performed in 1974 demonstrated that the weak Raman scattering of pyridine applied on the roughened silver surface could be enhanced by up to 6 orders of magnitude, and such a modified technique was called Surface-enhanced Raman spectroscopy (SERS) [23]. As early as 1980, scientists began introducing this technique to solve problems in biophysics and biochemistry, such as in research on peptides, pyrimidines, purines, and nucleic acids [24,25,26]. SERS holds great potential as a selective and specific technique where the signal of water is extremely weak. Taking advantage of all mentioned properties, the application of SERS has been extended to study microorganisms, e.g., bacteria [27,28], viruses [29,30], and fungi [31,32]. The information-rich spectra that can be obtained even from a single cell represent structural variations in the biochemical composition of investigated microorganisms. Indeed, the outer structure of microorganisms plays a significant role in pathogenesis, and knowing it may help to explain it. As SERS responses reflect the structural variations between analyzed microorganisms, it seems to be a perfect technique for identification purposes. The analysis of microorganisms by the Raman method was of interest in the early 2000s. For the first time, Maquelin et al. successfully utilized Confocal Raman Microspectrometry and supervised as well as unsupervised chemometric methods to discriminate between five species of *Candida* spp. and test the predictive abilities of created models [33]. Recently, the potential of SERS has been revealed to investigate microorganism studies aimed at (i) characterization, identification, and differentiation between some species of *Candida* spp. [34,35,36]; (ii) development of biomolecular sensing technology for *Candida albicans* identification [37]; (iii) characterization and monitoring of biofilm formation (*C. glabrata*, *C. albicans*) [38]; (iv) examination of the impact of surface structure on biofilm formation (*C. albicans*) [39]; (v) the direct detection of *Candida* spp. by capturing them on Fe_3_O_4_@PEI; (vi) the effect of concentration of Ag NPs and Au NPs on bacterial cells (*Lactobacillus fermentum*) in terms of toxicity [40].

In the present work, we demonstrate the application of SERS supported by the chemometric method—Partial Least Square Regression (PLSR)—to discriminate between commensals microorganisms (*Lactobacillus* spp., *Bifidobacterium* spp.) and pathogens (fungi such as *Candida* spp. and bacteria (*Streptococcus agalactiae*, *Gardnerella vaginalis*, *Atopobium vaginae*, *Prevotella bivia*, *Finegoldia magna*, *Mobiluncus mulieris*, *Mobiluncus curtisii*, *Aerococcus tetradius*, *Anaerococcus christensenii*) that may be found in vaginal microbiota. For the first time, spectral characterization was also conducted for the anaerobic/microaerophilic protist parasite *Trichomonas vaginalis*. We also determined the spectral differences between analyzed species that directly refer to the typical compounds in their structure. All these SERS data were proceeded by PLSR so that even the smallest changes would be clearly presented. This research enriches the number of spectral signatures in a SERS library of microorganisms being created by our group.

## 2. Results and Discussion

**Figure 1:**
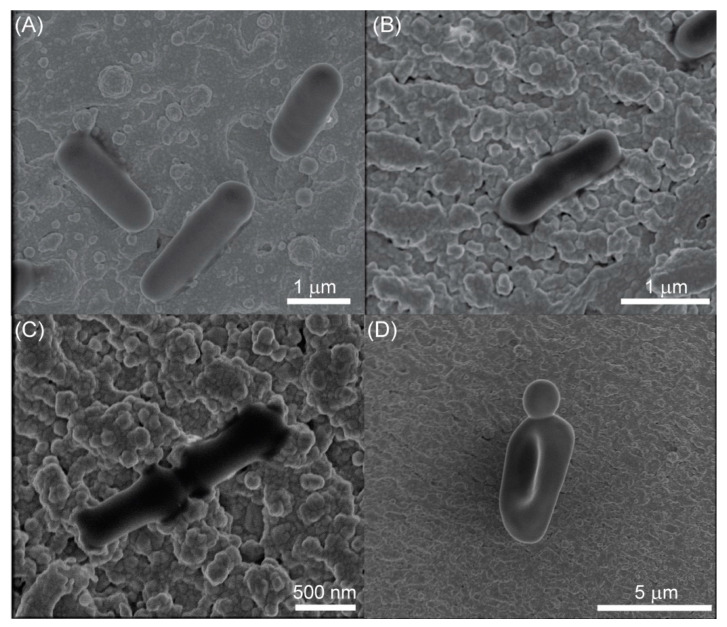
The SEM images of (**A**) *Lactobacillus fermentum*, (**B**) *Bifidobacteium longum*, (**C**) *Prevotella bivia*, and (**D**) *Candida albicans* dHp17 on the surface of the Si/Ag SERS platforms.

Recently, we determined the SERS profiles of gonococci—one of the most prevalent pathogens of the human urogenital tract [41]. This study aimed to record SERS signatures of bacteria (natural and causing BV), fungi, and protista—*T. vaginalis*—that may also be found in a human vagina and cause vaginal dysfunctions or STDs other than gonorrhea. These SERS measurements were assisted by Partial Least Squares Regression in order to find correlations between them in the associations as follows: (a) differentiation between *Candida* spp.; (b) differentiation between *Lactobacillus* spp.; (c) differentiation between *Bifidobacterium* spp.; (d) differentiation between *Lactobacillus* spp. and *Bifidobacterium* spp.; (e) differentiation between vaginal bacteria associated with BV (*P. bivia*, *F. vaginae*, *M. mulieris*, *M. curtisii*, *G. vaginalis*, *F. magna*, *A. tetradius*, *A. christensenii*) on MRS, RCM, TSA, and chocolate agar; (f) differentiation between strains of *Lactobacillus* spp., *Bifidobacterium* spp., and some microorganism found during vaginal dysbiosis or infection, namely *F. vaginae*, *P. bivia*, *G. vaginalis*, *C. glabrata*, and *C. albicans SN 128.* PLSR gives insight into specific variables responsible for the separation so that the recognition and identification of marker bands characteristic for analyzed association and/or species and/or microorganism can be achieved. As SERS platforms, we used silicon wafers surface-modified by laser ablation and covered with 100 nm of silver. Their significant advantage is that the fabrication process is ultrafast and straightforward and does not require chemical reagents. As this process involves a femtosecond laser working with high precision, the surface is highly uniform, ensuring the repeatability of the obtained signal [42]. The SEM images of analyzed microorganisms that reveal their shape, size, and location on SERS substrates are presented in Figure 1 and Figure S1. As can be seen, cells of *Lactobacillus* spp. [43], *Bifidobacterium* spp. [44], and *P. bivia* [45] are in the shape of rods. The nature of *C. albicans* is to create different forms: unicellular buddy yeast, hyphae, and pseudohyphae, a transition state between these two basic forms [46]. In Figure 1C, one can see pseudohyphae, where the daughter bud elongates. 

### 2.1. SERS-PLSR Analysis of Candida spp.—Vulvovaginal candidiasis (VVC) Disorder

**Figure 2:**
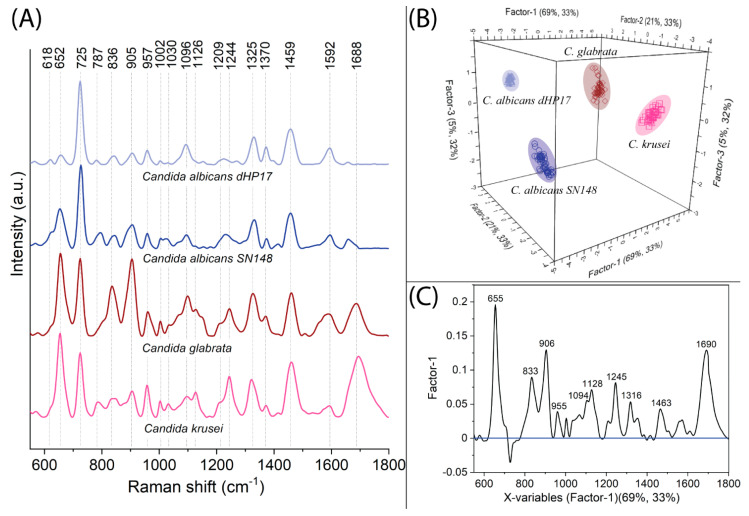
The SERS spectra (**A**) and PLSR results in the form of (**B**) 3D score plots and (**C**) X−loadings plots calculated for *Candida* spp.

Figure 2A presents the averaged SERS spectra of chosen *Candida* spp. often involved in candidiosis of the vagina, namely *C. glabrata*, *C. krusei*, *C. albicans SN148*, and *C. albicans BWP17*. Each spectral signature reveals bands that can be assigned to a specific oscillation of some biochemical compounds present in the outer structure of microorganisms. As SERS spectroscopy is very sensitive in terms of the quantity and quality of compounds, the spectral signature of every microorganism is unique.

The analysis of SERS spectra for *Candida* spp. clearly indicates that the most characteristic band is located at 730 cm^−1^ and originated from adenine-related compounds (FAD, NAD, ATP, DNA), as has already been proven [47]. Additional bands of great importance are 1096 cm^−1^ and 1126 cm^−1^, which come from branched chains of 1,3-β-D-glucan and 1,3-β-D-glucan and/or *N*-acetylglucosamine, respectively. These compounds constitute the building blocks of chitin and glucagon—polysaccharides that create most of the cell wall [36,48]. This was also proved by Noothalapati et al., who performed a spectral investigation of a distinct region of fungal cells—cytoplasm, cell wall, and lipid droplets. In his work, he recognized that the mentioned characteristic bands in the region 1000–1200 cm^−1^ originated from polysaccharides [49]. Other bands that indicate the presence of chitin and glucagon in the fungal structure are: 905, 954, 1209, 1244, 1325, 1370, 1459 cm^−1^. Mannoproteins (glycoproteins glycosylated with mannose) are the second major component of the fungal cell wall, accounting for 30–50% of its total content. Their presence can be manifested by the amide I and amide III bands 1244, 1592, and 1688 cm^−1^, and also 1325 and 1449 cm^−1^ [50]. Another band at 654 cm^−1^, which is extremely intensive for *C. krusei* and *C. glabrata*, can occur due to C-S stretching, C-C twisting of proteins, and/or COO^−^ deformation in amino acids. The bands that can be effective indicators in differentiation of *C. krusei* and *C. glabrata* against both strains of *C albicans* are located at 1126 and 1209 cm^−1^. *C. glabrata* can be additionally distinguished from *C. krusei* by the bands at 834 cm^−1^ and 905 cm^−1^ with the relative intensity ratio equaling I_854_ = 2.7 and I_905_ = 2.9, respectively. A key aspect is that every *Candida* spp. reveals bands at 834 cm^−1^, 905 cm^−1^, 1209 cm^−1^, and 1370 cm^−1^ with a significant intensity that allows for the distinction of the fungi from bacteria analyzed here. Bacteria are usually characterized by a lack of these bands or their negligible intensity (see Table 1).

The results of the PLSR analysis conducted for this association indicate that the four species of *Candida* can be differentiated by three main factors. Factor-1 always explains the maximum information, and in this case, it is 76% of the variance in block Y with 36% of data within the X matrix. As can be empirically estimated from SERS spectra, *C. albicans* SN 148 and *C. albicans BWP17* are remarkably similar to each other in comparison to the rest of the species. This dependency is visualized by the PLS score, as the Factor-1-located *C. albicans SN148* and *C. albicans BW17* form the *C. glabrata* and *C. krusei* in a large distance (Figure 2B). The X-loadings plot specifies the variables crucial for segregation within the analyzed group of data (Figure 2C). Hence, in the association of *Candida* spp. the surface proteins (e.g., mannoproteins) have a very significant impact on differentiation—654, 834, 1459, and 1694 cm^−1^. Since the weight of variables 906, 955, 1096, 1128, 1244, and 1316 cm^−1^ have a high value, the concentration of chitin and glucan (polysaccharides) varies between species; hence, they can also be used as diagnostic markers.

### 2.2. SERS-PLSR Analysis of Lactobacillus spp. and Bifidobacterium spp.—Natural Vaginal Flora

**Figure 3:**
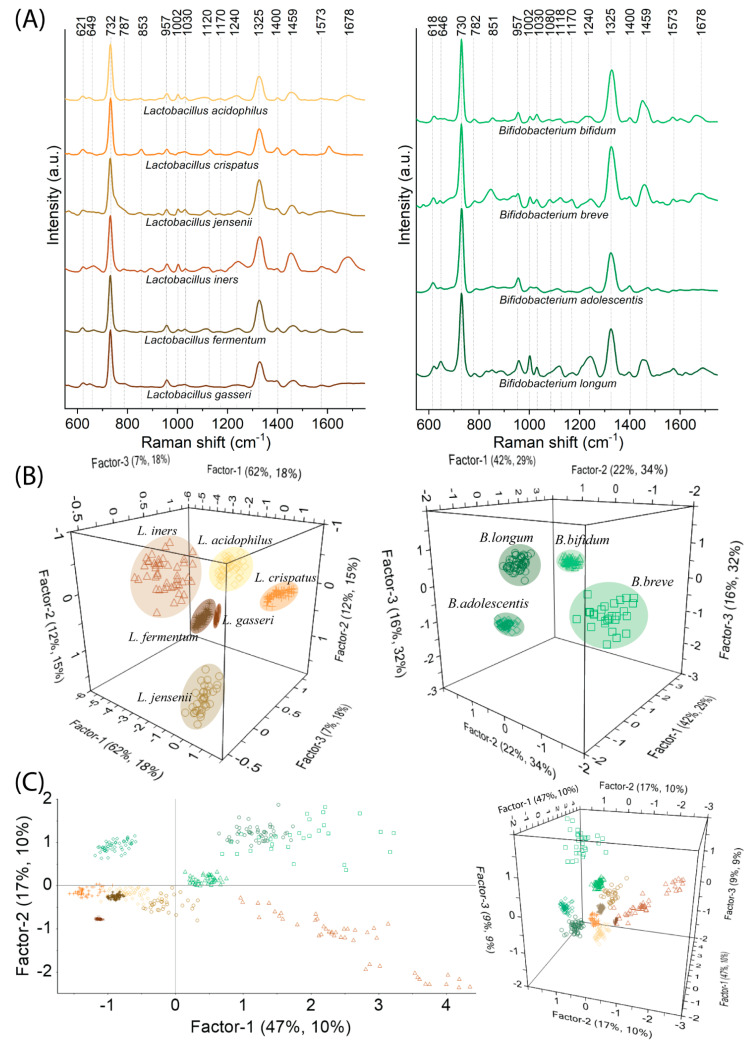
The SERS spectra for *Lactobacillus* spp. and *Bifidobacterium* spp. (**A**) and PLSR results in the form of score plots calculated for the following associations: (**B**) *Lactobacillus* spp. and *Bifidobacterium* spp. separately (inter-species distinction) and (**C**) *Lactobacillus* spp. and *Bifidobacterium* spp. together (intergeneric distinction).

As was mentioned above, *Lactobacillus* spp. and (in some cases) *Bifidobacterium* spp. constitute the dominant part of the vaginal microflora and guarantee protective and even health-promoting effects [8]. Figure 3A presents SERS spectra of the most significant *Lactobacillus* spp. and *Bifidobacterium* spp. and their PLSR analysis performed in two associations: (i) *Lactobacillus* spp. and *Bifidobacterium* spp. separately (inter-species distinction) (Figure 3B); (ii) *Lactobacillus* spp. and *Bifidobacterium* spp. together (intergeneric distinction) (Figure 3C). All these spectra reveal many similar bands; the most significant are located at 731, 1325, and 1459 cm^−1^ and indicate the presence of adenine, proteins, and lipids, respectively. As empirical observations and analyses are quite problematic and inaccurate, the PLSR analysis calculated for particular associations brings many advantages and gives us deep insight into the spectral differences. First of all, PLSR scores for both associations present very well separated groups of species. For *Lactobacillus* spp. the Factor-1 explains 62% of variance in block Y with 18% of data within the X matrix (Figure 3B), while for *Bifidobacterium* spp. Facor-1 explains 42% of Y variance with 29% of X variance (Figure 3B). Calculated X-loadings plots for these two associations that correspond to Factor-1 are presented in Figure S2A,B. For *Lactobacillus* spp. as well as *Bifidobacterium* spp. variables at 1002, 1124, 1251, 1334, and 1452 cm^−1^ have significant values. Another intense marker band at 1681 cm^−1^ indicates that the concentration of proteins (especially for *Lactobacillus* spp.) varies between analyzed species, while the significant variations in tyrosine, nucleic acids (guanine, thymine), lipids, and phospholipids documented by 647, 845, 1080, and 1168 cm^−1^ are additional markers for *Bifidobacterium* spp. differentiation. All these mentioned and other (even slight) changes determine that the differentiation at the species level among *Bifidobacterium* spp. and *Lactobacillus* spp. is achievable (Figure 3B).

Despite the fact that it is hard to empirically designate some distinctive features for *Lactobacillus* spp. and *Bifidobacterium* spp. that would ambiguously indicate the membership of particular bacteria to the correct group (whether it is *Lactobacillus* or *Bifidobacterium*), such designation is possible with PLSR analysis (Figure 3C). Factor-1 explains most of the spectral information accounting for 46% of Y variance and 10% of X spectral variance. At the same time, Factor-2 designates the boundaries between all analyzed *Lactobacillus* spp. and *Bifidobacterium* spp. encompassing 15% of the variance in block Y with 10% of data within the X matrix. From the X-loadings plot calculated for this association (Figure S2C), we find that variables for Factor-1 are consistent with the variables for *Lactobacillus* spp. and *Bifidobacterium* spp. For Factor-2 the variables with positive values refer to the group of *Bifidobacterium* spp. and include 613, 644, 843, 946, 1002, and 1317 cm^−1^, while those with negative values—1271, 1361, 1454, and 1668 cm^−1^—refer to *Lactobacillus* spp. The variables mentioned above reflect all these biochemical changes in outer structures typical for these two species individually and provide their effective discrimination. Hence, the variation in the content of (*i*) phenylalanine, tyrosine, and nucleic acids for *Bifidobacterium* spp. and (*ii*) proteins and lipids for *Lactobacillus* spp. are responsible for their binary discrimination and this specific allocation along Factor-2. In the comparison of *Candida* spp. vs. *Lactobacillus* spp. and *Bifidobacterium* spp. some additional bands such as 850, 1080, 1170, and 1400 cm^−1^ characterize only bacteria. Since their intensity is low (for some species, it is even neglected), considering them as marker bands is questionable at this stage of the analysis.

### 2.3. SERS-PLSR Analysis of the Most Prevalent Bacteria Responsible for BV

**Figure 4:**
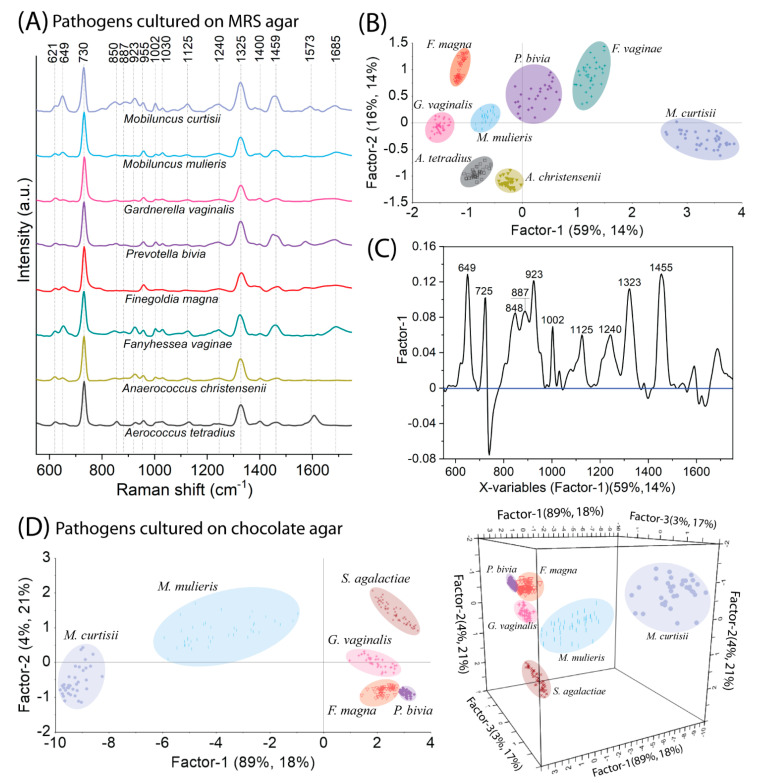
The SERS spectra (**A**) and PLSR results in the form of score plot (**B**) and X−loadings plot (**C**) for the most prevalent pathogenic bacteria cultivated on MRS agar, and PLSR results in the form of score plots 2D and 3D (**D**) for pathogenic bacteria cultivated on chocolate agar.

The most prevalent bacteria that cause BV are: *M. mulieris*, *M. curtisii*, *G. vaginalis*, *F. magna*, *P. bivia*, *F. vaginae*, *A. christensenii*, *A. tetradius*. As a first step, we standardized a procedure to ensure the best growth conditions (selecting an appropriate culture medium among MRS, TSA, and RCM) and, more importantly, the maximal differences between spectral responses for cultured microorganisms. For this purpose, bacteria were streaked on appropriate media, their growth quality was assessed, their SERS spectra were measured, and then PLSR analysis was performed for appropriate associations—MRS, RCM, TSA medium—so that their spectral differences could be determined. Figure S3a,b present the PLSR score plots for mentioned pathogens grown on RCM and TSA medium and Figure 4A–C present the results for all of them grown on MRS medium. The spectra of *F. magna* and *P. bivia* grown on TSA medium cannot be separated enough by the PLSR method, which obviously indicates faint spectral differences between them. In turn, *P. bivia* could not be grown on RCM. Based on the obtained results, we conclude that these two media (TSA and RCM) do not work effectively enough. The MRS medium provided the weakest growth of all microorganisms. Although the composition of the MRS medium is not best suited to the requirements of the bacteria, it provided enough growth for all of them to the spectral differences significant enough so that all species are well separated in the PLSR dimension (Figure 4B detailed explanation below).

Preliminary analysis of the obtained results (Figure 4A) indicates that the spectral variations are particularly concerned with mutual differences in intensity ratios of the individual bands (e.g., 649, 925, and 1459 cm^−1^). Empirically we observe that *M. curtisii* is recognized by the intense bands in the region 850–955 cm^−1^ and one located at 649 cm^−1^. At the same time, *F. magna* and *G. vaginalis* can be distinguished from the rest of the studied microorganisms by weak bands almost in all spectral ranges. The uniqueness of spectral responses for each microorganism can be confirmed by the fact that Factor-1 explains 14% of the variance in the block Y with 59% of the spectral data (X matrix), and visually this can be presented by well-resolved groups on PLSR score as was mentioned already (Figure 4B). The X-loadings plot indicates that most bands that mainly differ between bacteria are 646, 848, 887, 923, 1002, 1125, 1240, 1325, and 1445 cm^−1^ that have arisen from structural changes mainly between *P. bivia*, *F*. *vaginae*, and *M. curtisii* (Figure 4C).

As was mentioned, *S. agalactiae* is an important pathogen that poses a threat to newborns, and therefore should not be omitted from consideration. Since *S. agalactiae* grows in a chocolate medium, it is reasonable from the diagnosis point of view to compare it only with bacteria that are equally able to be grown on the same medium. These bacteria include: *F. magna*, *G. vaginalis*, *P. bivia*, *M. mulieris*, *M. curtisii.* The PLSR algorithm provides an excellent distinction between analyzed bacteria. Factor-1 explains 18% of the variance in the Y block with 89% of the original spectral data and divides the group of *M. mulieris* and *M. curtisii* from the rest of the bacteria (Figure 4D). *S. agalactiae* is relatively further allocated than the other bacteria due to several prominent spectral features such as: a very intense band at 649 cm^−1^, an additional one at 1180 cm^−1^, and the one that is shifted from 1240 to 1220 cm^−1^ (Figure S4).

### 2.4. Bacterial Vaginosis vs. Vulvovaginal Candidiasis—SERS-Based Diagnosis

**Figure 5:**
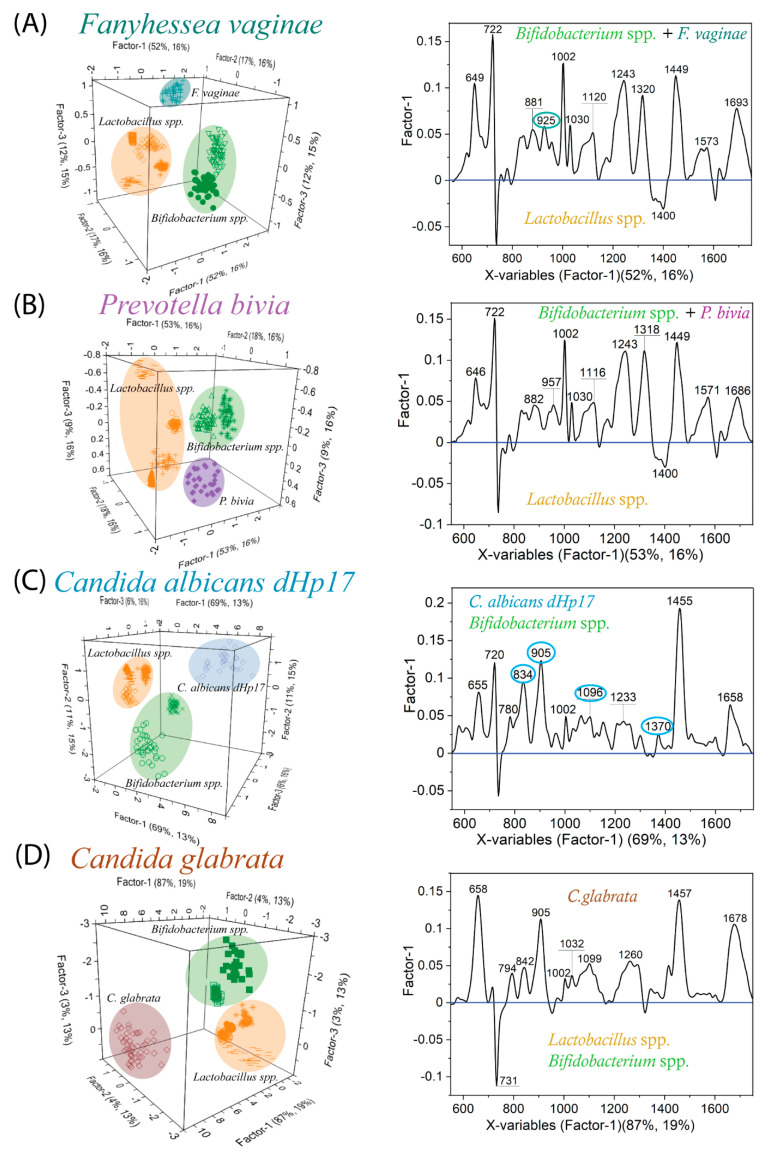
The results of PLSR analysis in the form of score plots and X−loading plots for various associations such as 4 chosen strains of *Lactobacillus* spp., 2 chosen strains of *Bifidobacterium* spp., and *F. vaginae* (**A**), *P. bivia* (**B**), *C. albicans dHp17* (**C**), *C. glabrata* (**D**). All microorganisms were cultivated on MRS agar for 48 h.

In order to examine the applicability of the SERS-PLSR approach for diagnostic purposes, the different associations that contain the following microorganisms were examined: fungi (*C. glabrata*, *C. albicans dHp17*), bacteria from *Lactobacillus* spp. *Bifidobacterium* spp., and pathogens (*G. vaginalis*, *F. vaginae*, *P. bivia*). Significantly, all these bacteria and fungi that were taken into calculations were cultivated on MRS agar for 48 h. Thus, in actual diagnostic conditions, they may be confusing. Figure 5A–D present the results of PLSR calculations performed in five different sets—always four strains of *Lactobacillus* spp., two strains of *Bifidobacterium* spp., and one of the pathogens (*C. glabrata*, *C. albicans dHp17*, *F. vaginae*, *P. bivia*) were chosen. For each case under consideration, all species are well separated in the PLSR dimension, and the obvious grouping of species within the same genre can be noticed, concerning clusters of *Lactobacillus* spp., *Bifidobacterium* spp., and every pathogen that does not overlap with the other clusters. From analyzed PLSR scores, it follows that for associations with *F. vaginae* and *P. bivia* (Figure 5A,B), Factor-1 allocates *Lactobacillus* spp. on the negative side of the score plot against *Bifidobacterium* spp. together with the considered pathogen on the opposite side (positive values). The subsequent factors describe all spectral data at a similar level. For better visualization, Table S1 gathers the percentage of explained information for the major factors. Calculated X-loadings plots (Figure 5A,B) indicate that the variable at 1400 cm^−1^ with a negative value comes from *Lactobacillus* spp., while the ones with positive values which include mainly ~649, 1002, 1243, 1320, and 1445 cm^−1^ correspond to variations between spectra of *Bifidobacterium* spp. and pathogens (*F. vaginae*, or *P. bivia*), giving significant contribution for such separation. Prominently, such analysis disclosed a specific band at 923 cm^−1^ (marked in a turquoise circle in Figure 5A) which, due to being typical of *F. vaginae*, may represent a relevant marker band in this association. Since it was reasonable for a mixed comparison involving fungi and bacteria to maintain the same growth conditions, Figure S5 shows the spectral images for the two considered strains—*C. glabrata* and *C. albicans dHp17*—cultured on MRS agar for 48 h. Taking into account the possibilities and extreme sensitivity of the SERS technique, we supposed that the spectral changes resulting from the time of cultivation and the nutrients of the culture medium might be meaningful. As the comparative analysis (Figure 2A and Figure S5) demonstrates, the alterations in growth conditions affect the spectral changes in the form of, e.g., differences in intensities of bands 725 and 1455 cm^−1^, new bands (1400, 1060 cm^−1^ for *C. albicans dhP17*) that can be empirically designated.

Nonetheless, all these marker bands describing the yeast were retained, the same as for the YPD that grew for 24 h. For the association (Figure 5C), Factor-1 allocates *Lactobacillus* spp. (negative scores) against *Bifidobacterium* spp. together with *C. albicans dHp17* (positive scores), which is considerably distant from the rest of the bacteria. The X-loadings plot reveals the fungal marker bands 834, 905, 1096, and 1370 cm^−1^ (marked in blue circles in Figure 5C). The most significant variable is located at 1455 cm^−1^, and such a significant contribution is mainly due to *C. albicans dHp17*. The rest of the variables have origins in both fungus and *Bifidobacterium* spp. In such a comparison, no contribution from them was noted because of the neglectable Lactobacillus’s band intensities (no negative variables). The best separation can be obtained while comparing *Lactobacillus* spp. *Bifidobacterium* spp. with *C. glabrata* (Figure 5D). Factor-1 that explains 19% of the Y matrix with 87% of original data significantly differentiates fungi (positive scores values) from the rest of the bacteria (negative scores values). The corresponding X-loadings plot (Figure 5D) designates that all the variables with positive weights refer to the spectrum of *C. glabrata*, and the variable at 724 cm^−1^ (negative weight) documents the contribution of bacteria in the entire distinction. As can be concluded from the aforementioned consideration, this analysis reveals that the band at 1400 cm^−1^, even at low intensity, can be a marker band for *Lactobacillus* spp. in some associations.

### 2.5. The Spectral Fingerprint of Trichomonas vaginalis—A Parasite That Leads to Trichomoniasis

**Figure 6:**
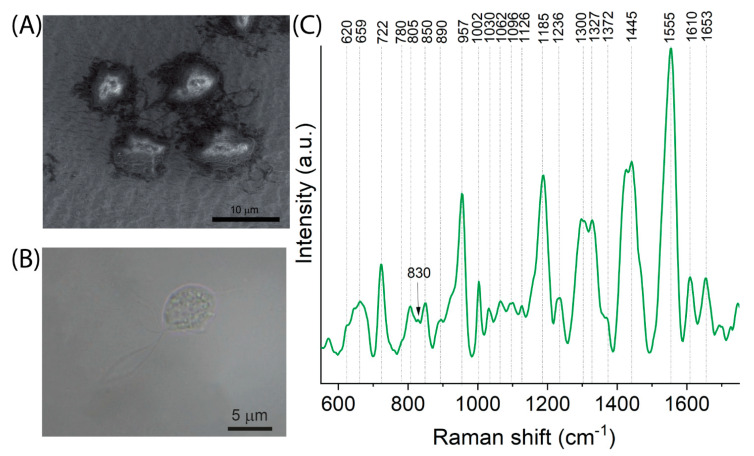
Results of *Trichomonas vaginalis* analysis: (**A**) SEM images that present cells of *T. vaginalis* located on SERS substrates and (**B**) microscopic image of *T. vaginalis* cell and (**C**) averaged SERS spectrum.

Figure 6 shows the results of the spectroscopic and microscopic analysis performed for *T. vaginalis*. Figure 6A presents SEM images of *Trichomonas vaginalis* cells located on SERS substrates and the view of its cells under an optical microscope (Figure 6B). The analysis clearly shows that *T. vaginalis* cells are pear-shaped, and their dimensions are within the range between 10 μm and 45 μm. Moreover, such microscopic analysis reveals the structure of the protozoan consisting of four anterior flagella, one posterior flagellum, and an undulating membrane arranged along the body of the protozoan cell that enables its quivering motility and cytoplasm with the nucleus, granules, spare bodies with glycogen, and volutin.

The spectral analysis of *T. vaginalis* cells indicates the presence of proteins (957 cm^−1^, 1442 cm^−1^), lipids (1185 cm^−1^, 1442 cm^−1^), and purine bases adenine and polyadenine (722 cm^−1^, 1327 cm^−1^). The outer structure of protozoan consists of LPG (lipophosphoglycan) with poly-N-acetyllactosamine repeats, which are rich in lipids and carbohydrate components. This part is anchored via phospho-inositol ceramide (CPI) to the cell surface [51]. Consequently, we observe typical bands at 1062, 1126, 1300, and 1653 cm^−1^ that come from ceramides [52] and a lipid moiety and 850, 890, 1030, 1062, 1096, 1126, and 1370 cm^−1^, indicative of saccharides/carbohydrates [53]. *T. vaginalis*, through the production of proteins from the adhesin group, are able to attack host cells which are manifested by the bands located at 659, 890, 957, 1236, 1444, 1555, and 1653 cm^−1^ [53]. Empirical analysis of all the spectral data considered in this study demonstrates that marker bands of *T. vaginalis* are located at 805, 890, 1062, 1185, 1300, 1555, and 1610 cm^−1^.

## 3. Materials and Methods

### 3.1. Bacteria Species and the Culture Conditions

The following bacteria strains, *Lactobacillus gasseri* PCM 2500, *Lactobacillus fermentum* PCM 491, and *Lactobacillus acidophilus* PCM 2499, were obtained from the Polish Collection of Microorganism (PCM) of the Institute of Immunology and Experimental Therapy (Wroclaw, Poland). In turn, *Lactobacillus crispatus* 30722T, *Lactobacillus iners* 28746T, *Lactobacillus jensenii* 35572T, *Bifidobacterium breve* 30511AT, *Bifidobacterium longum* 28903T, *Bifidobacterium bifidum* 45217T, *Bifidobacterium adolescentis* 17359T, *Prevotella bivia* 9557T, *Fanyhessea vaginae* 38953T, *Mobiluncus mulieris* 20071T, *Mobiluncus curtisii* 21018T, *Gardnerella vaginalis* 3717T, *Finegoldia magna* 17636T, *Aerococcus tetradius* 46590T, and *Anaerococcus christensenii* 28831T were obtained from the Culture Collection of the University of Gothenburg (CCUG). Fungi, *Candida glabrata*, *Candida krusei* 74256, *Candida albicans SN148*, and *Candida albicans BWP17* were obtained from the CCUG or the Institute of Biochemistry and Biophysics (IBB) collection (Warsaw, Poland). *Trichomonas vaginalis* (ATCC-30001) was obtained from American Type Culture Collection (ATCC, USA). The list of growth conditions and culture media selected for the analyzed microorganisms is presented in Table S2.

### 3.2. Sample Preparation for SERS Measurements

Each colony of bacteria was carefully taken via inoculation loop and placed into 100 μL of 0.9% NaCl solution and mixed to obtain a turbid suspension. Then, the solution was centrifuged for two minutes at 1070× *g*. The supernatant was discarded, and the next portion of saline solution was added again. In order to obtain a purified solution of bacterial cells, this operation was repeated three times. After this, the pellet was resuspended in 15 μL of NaCl solution. Next, 1 μL of bacterial cell solutions was placed over freshly prepared SERS substrate and left to dry for a few minutes under a laminar flow cabinet.

### 3.3. SERS Substrate Preparation

The SERS-active silicon-based substrates covered with silver (the Si/Ag SERS platforms) were prepared in a few steps. First, a clean silicon wafer was mechanically cut into 3 mm × 3 mm pieces. Then, the substrates were physically subjected to laser ablation with a femtosecond laser, where the parameters were as follows: wavelength λ = 1030 nm, the repetition rate 300 kHz, and pulse width of 300 femtoseconds. Finally, they were covered with a 100 nm layer of silver via a Physical Vapor Deposition (PVD) device (Quorum, model Q150T ES, Laughton, UK). The procedure was described in detail by Szymborski et al. [42].

### 3.4. SERS Spectroscopy and Chemometric Analysis

Measurements were performed by using Bruker’s BRAVO spectrometer equipped with Duo LASER™ (700 nm–1100 nm) and a CCD camera. The spectral resolution was 2–4 cm^−1^, while the laser power was 100 mW for both LASERs. The background was automatically removed. To demonstrate the reproducibility of our experiments, SERS measurements were recorded repeatedly to obtain 50 single measurements for each tested bacterial strain. The recording time of a single spectrum was about 6000 ms and 3 accumulations. Using OPUS software (Bruker Optic GmbH, 2012 version, Leipzig, Germany), all obtained spectra were processed: baseline correction (concave rubber band correction, number of iterations 6, number of baseline points 6), smoothing (number of smoothing points 5), normalization (min-max normalization, the whole range) and then cutting in the range between 600 cm^−1^ and 1750 cm^−1^. Finally, all the preprocessed data were put into Unscrambler (CAMO software AS, version 10.3, Oslo, Norway), where the Partial Least Square Regression (PLSR) was performed.

### 3.5. Microscopic Characterization

To characterize the morphology of the bacteria, yeast, and other pathogens (*Trichomonas vaginalis*), we used optical microscopy and scanning electron microscopy (SEM).

SEM measurements were performed under a high vacuum using the FEI Nova NanoSEM 450 (Hillsboro, OR, USA). The accelerating voltage was in the range of 2 kV to 10 kV. The SERS platforms were placed on the SEM stubs using a carbon or silver conductive paste (Ted Pella, Redding, CA, USA). No additional layer of carbon or gold was used on the surface of the sample to improve the conductivity.

Observations with optical microscopy were conducted using an upright microscope Delta Optical Evolution 100 Trino Plan LED (Delta optical, Poland). The microscope was equipped with a ×10 eyepiece and a set of objective lenses: ×4, ×10, ×40, and ×100 (for immersion). The ×100 lens was used with cedar immersion oil (Merck, Darmstadt, Germany). The microscope was equipped with a camera port and Canon-compatible adapter. For the acquisition of the images, we used a DSLR camera, Canon 40D (Canon, Japan), connected with a laptop. The recording of the images and HD videos at 60 fps was conducted by Canon EOS Utility software. RAW files were developed with ON1 Photo RAW software (ON1, Portland, OR, USA).

## 4. Conclusions

The results presented in this study raised an important issue concerning various types of microorganisms (bacteria, fungi, protozoa) forming vaginal microbiota or being pathogens that have a positive as well as negative impact on women’s health. The accurate differentiation between them is crucial and finding an appropriate method for such discrimination is extremely necessary. In this study, we showed that each microorganism reveals a unique spectral fingerprint, and with the support of PLSR analysis, the discrimination between them can be achievable. A complete comparison of these three types of microorganisms—fungi, bacteria, and protozoa—and their designated marker bands is the following: fungi 905 and 1209 cm^−1^, *Lactobacillus* spp. 1400 cm^−1^, protozoa 805, 890, 1062, 1185, 1300, 1555, and 1610 cm^−1^. Even though it is hard to empirically observe the differences among *Lactobacillus* spp. and *Bifidobacterium* spp., PLSR deals well with this problem and provides the differentiation between genres along Factor-2. Moreover, the discrimination while comparing *Lactobacillus* spp., *Bifidobacterium* spp., and some pathogens is also possible with a high level of information explained by subsequent factors. The discrimination among pathogens responsible for BV was also achieved with the information at the level of 56% of the Y matrix and 96% of the X matrix, explained by the first three factors. These studies are a necessary part of the subsequent research involving clinical samples.

## Figures and Tables

**Table 1:** The tentative assignments for the most prominent bands of bacteria, *Candida* spp. and *Trichomonas vaginalis* analyzed in the present study. Compounds typical for a specific group of microorganisms are bolded. Ranges of spectral bands of different bacteria species are distinguished by symbols: ^a^ for *Lactobacillus* spp., ^b^ for *Bifidobacterium* spp., ^c^ for pathogens grown in MRS agar, ^d^ for pathogens on RCM agar, ^e^ for pathogens on TSA agar, and ^f^ for pathogens on chocolate agar.

Raman Shifts (cm^−1^) for *Candida* spp.	Assignment for *Candida* spp.	Raman Shifts (cm^−1^) for Bacteria	Assignment for Bacteria	Raman Shifts (cm^−1^) for *T. vaginalis*	Assignment for *T. vaginalis*
618	C-C twisting mode of phenylalanine (protein)	621 ^a^ 620 ^b^ 622 ^c^ 622 ^d^ 624 ^e^ 622 ^f^	C-C twisting mode of phenylalanine (protein)	620	C-C twisting mode of phenylalanine (protein)
654	C-S stretching, C-C twisting of proteins (tyrosine) COO^−^ deformation in amino acids, guanine and thymine (ring breathing modes)	649–664 ^a^ 645–652 ^b^ 649 ^c^ 649–665 ^d^ 647 ^e^ 649 ^f^	C-S stretching, C-C twisting of proteins (tyrosine) COO^−^ deformation in amino acids, guanine and thymine (ring breathing modes)	659	C-S stretching, C-C twisting of proteins (tyrosine) COO^−^ deformation in amino acids, guanine and thymine (ring breathing modes)
725	Adenine (FAD, NAD, ATP, DNA)	732 ^a^ 730 ^b^ 730 ^c^ 730 ^d^ 730 ^e^ 730 ^f^	Adenine (FAD, NAD, ATP, DNA)	722	Adenine (FAD, NAD, ATP, DNA)
787	Ring breathing mode of cytosine, uracil, thymine, (O−P−O) symmetric stretching of nucleic acid	782–787 ^a^ 781–794 ^b^ 790–800 ^c^ 790–800 ^d^ 787 ^e^ 784–791 ^f^	Ring breathing mode of cytosine, uracil, thymine, (O−P−O) symmetric stretching of nucleic acid	780	Ring breathing mode of cytosine, uracil, thymine, (O−P−O) symmetric stretching of nucleic acid
-	-	-	-	805	Uracil-based ring breathing mode
834	Stretching O-P-O of DNA deformative vibrations of amine groups, tyrosine	-	-	830	Stretching O-P-O of DNA, deformative vibrations of amine groups, tyrosine
-	-	847–855 ^a^ 846–853 ^b^ 845–847 ^c^ 853 ^d^ 853 ^e^ 853 ^f^	Thymine, ring breathing mode of tyrosine, C-C stretch of proline ring, single bond stretching vibrations for the amino acids and valine and polysaccharides	850	Thymine, ring breathing mode of tyrosine, C-C stretch of proline ring, single bond stretching vibrations for the amino acids and valine and polysaccharides
-	-	-	-	890	Proteins, saccharides
905	Chitin, monosaccharide (B glucose) C-C skeletal stretching, tyrosine	-	-	-	-
957	Chitin, C=C deformation, C-N stretching, C−O stretching, CH_3_ symmetric stretching of proteins (α-helix)	957 ^a^ 956 ^b^ 955 ^c^ 956 ^d^ 956 ^e^ 956 ^f^	C=C deformation, C-N stretching, C−O stretching, CH_3_ symmetric stretching of proteins (α-helix)	957	C=C deformation, C-N stretching, C−O stretching, CH_3_ symmetric stretching of proteins (α-helix)
1002	Phenylalanine, C-C aromatic ring stretching	1002 ^a^ 1002 ^b^ 1002 ^c^ 1002 ^d^ 1002 ^e^ 1002 ^f^	Phenylalanine, C-C aromatic ring stretching	1002	Phenylalanine, C-C aromatic ring stretching
1030	C-N stretching, C-C stretching (phospholipids, carbohydrates e.g., chitin), C-H in-plane bending mode of phenylalanine	1030 ^a^ 1030 ^b^ 1030 ^c^ 1030 ^d^ 1030 ^e^ 1030 ^f^	C-N stretching, C-C stretching (phospholipids, carbohydrates), C-H in-plane bending mode of phenylalanine	1030	C-N stretching, C-C stretching (phospholipids, carbohydrates), C-H in-plane bending mode of phenylalanine
-	-	-	-	1062	Acyl group of ceramides stretching O-P-O of DNA, skeletal C-C stretching (lipids), C-O, C-C stretching (carbohydrates)
1096	1,3-β-D-glucan, PO_2_^-^ symmetric stretching, C–O–C stretching modes in polysaccharides (e.g., chitin), adenine, polyadenine ν(C-C), ν(C-O), phospholipids	1070–1080 ^b^ 1072 ^c^ 1080–1096 ^d^ 1088–1097 ^e^ 1096 ^f^	PO_2_^-^ symmetric stretching, C–O–C stretching modes in polysaccharides, adenine, polyadenine, ν(C-C), ν(C-O), phospholipids	1096	PO_2_^-^ symmetric stretching, C–O–C stretching modes in polysaccharides, adenine, polyadenine, ν(C-C), ν(C-O), phospholipids
1126	=C-O-C= (unsaturated fatty acids in lipids), C-O-C stretching modes in nucleic acids, PO_2_^_^ stretching in nucleic acid, C-O and C-C stretching in carbohydrates, C-N stretching in proteins, 1,3-β-D-glucan, *N*-acetylglucosamine	1120 ^a^ 1112–1126 ^b^ 1124 ^c^ 1122 ^d^ 1124 ^e^ 1124 ^f^	=C-O-C= (unsaturated fatty acids in lipids), C-O-C stretching modes in nucleic acids, PO_2_^_^ stretching in nucleic acid, C-O and C-C stretching in carbohydrates, C-N stretching in proteins	1126	=C-O-C= (unsaturated fatty acids in lipids), C-O-C stretching modes in nucleic acids, PO_2_^_^ stretching in nucleic acid, C-O and C-C stretching in carbohydrates, C-N stretching in proteins Acyl group of ceramides
-	-	1170 ^a^ 1170 ^b^	C-H in-plane bending mode of tyrosine, ν(C=C) δ(COH) (lipid)	-	-
-	-	-	-	1185	Cytosine, guanine, adenine
1209	Chitin	-	-	-	-
1244	Amide III (arising from coupling of C-N stretching and N-H bonding—can be mixed with vibrations of side chains), β-1,3-glucan	1230–1248 ^a^ 1230–1248 ^b^ 1243 ^c^ 1240 ^d^ 1243 ^e^ 1245 ^f^	Amide III (arising from coupling of C-N stretching and N-H bonding—can be mixed with vibrations of sidechains)	1236	Amide III (arising from coupling of C-N stretching and N-H bonding—can be mixed with vibrations of side chains)
-	-	-	-	1300	CH_2_ deformation twisting and wagging (lipids), phospholipids, fatty acids, Acyl group of ceramides
1325	Chitin, β-1,3-glucan, mannose, CH_3_CH_2_ wagging in purine bases, NH_2_ stretching in adenine and polyadenine, phospholipids	1325 ^a^ 1325 ^b^ 1325 ^c^ 1325 ^d^ 1325 ^f^	CH_3_CH_2_ wagging in purine bases, NH_2_ stretching in adenine and polyadenine, phospholipids	1327	CH_3_CH_2_ wagging in purine bases, NH_2_ stretching in adenine and polyadenine, phospholipids
1370–1380	Chitin, 1,3-β-D-glucan, ring breathing modes of the DNA/RNA bases, COO^−^ stretching, C-H bending of proteins, saccharides	-	-	1372	Ring breathing modes of the DNA/RNA bases, COO^−^ stretching, C-H bending of proteins, saccharides
-	-	1400 ^a^ 1400 ^b^ 1400 ^c^ 1400 ^d^ 1400 ^e^ 1400 ^f^	C=O symmetric stretch, CH_2_ deformation, N-H in plane deformation	-	-
1459	Chitin, mannose, 1,3-β-D-glucan, CH_2_ deformation (protein, lipids), CH_2_ bending mode of protein and lipids, C-H vibrations proteins and lipids CH_2_ wagging, CH_2_/CH_3_ deformation, CH_2_ bending and scissoring of phospholipids	1449–1468 ^a^ 1449–1468 ^b^ 1449–1468 ^c^ 1449–1468 ^d^ 1449–1468 ^e^ 1449–1463 ^f^	CH_2_ deformation (protein, lipids), CH_2_ bending mode of protein and lipids, C-H vibrations proteins and lipids, CH_2_ wagging, CH_2_/CH_3_ deformation, CH_2_ bending and scissoring of phospholipids	1444	CH_2_ deformation (protein, lipids), CH_2_ bending mode of protein and lipids, C-H vibrations proteins and lipids, CH_2_ wagging, CH_2_/CH_3_ deformation, CH_2_ bending and scissoring of phospholipids
-	-	-	-	1555	Stretching C-N and deformation N-H of Amide II
1584–1598	Amide I band of proteins (mannans), due to C=O stretching C=C bending mode (phenylalanine), guanine, adenine, tryptophan	1570–1578 ^a^ 1570–1576 ^b^ 1573 ^c^ 1570 ^d^ 1590 ^e^ 1578 ^f^	Amide I band of proteins, due to C=O stretching C=C bending mode (phenylalanine), guanine, adenine, tryptophan	-	-
-	-	-	-	1610	Cytosine NH_2_, tyrosine, phenylalanine ring vibration
1663–1693	Amide I of proteins, ν(C=C) cis, lipids, fatty acids Carbonyl stretch (C=O)	1660–1680 ^a^ 1660–1690 ^b^ 1680–1689 ^c^ 1670–1700 ^d^ 1680–1700 ^e^ 1664–1694 ^f^	Amide I of proteins, ν(C=C) cis, lipids, fatty acids Carbonyl stretch (C=O)	1653	Ceramide backbone, Amide I of proteins, ν(C=C) cis, lipids, fatty acids Carbonyl stretch (C=O)

## Data Availability

Not applicable.

## Supplementary Materials

The supporting information can be downloaded at: https://www.mdpi.com/article/10.3390/ijms232012576/s1.
