# Supplementary material for: SERS-PLSR Analysis of Vaginal Microflora: Towards the Spectral Library of Microorganisms
Source: Int J Mol Sci. 2022 Oct 20;23(20):12576. doi: 10.3390/ijms232012576 (PMC9604117; doi:10.3390/ijms232012576)
Supplement: Supplementary file 1 [file ijms-23-12576-s001.zip › ijms-1979566-supplementary.pdf]

Supplementary information

SERS-PLSR Analysis of Vaginal Microflora: Towards the Spectral Library of Microorganisms

Sylwia Magdalena Berus [1,*,†], Monika Adamczyk-Popławska [2,†], Katarzyna Goździk [3], Grażyna Przedpełska [4], Tomasz R. Szymborski [1], Yuriy Stepanenko [1] and Agnieszka Kamińska [1,*]

[1] Institute of Physical Chemistry, Polish Academy of Sciences, Kasprzaka 44/52, 01-224 Warsaw, Po-land

[2] Department of Molecular Virology, Faculty of Biology, University of Warsaw, Miecznikowa 1, 02-096 Warsaw, Poland

[3] Department of Parasitology, Faculty of Biology, University of Warsaw, Miecznikowa 1, 02-096 Warsaw, Poland

[4] Department of Dermatology and Venerology, Infant Jesus Clinical Hospital, Koszykowa 82a, 02-008 Warsaw, Poland

* Correspondence: sberus@ichf.edu.pl (S.M.B.); akaminska@ichf.edu.pl (A.K.)

† These authors contributed equally to this work.

1. Tables

Table S1. The set of percentage explained information for three first components for different associations.

Association	Factor-1		Factor-2		Factor-3		Total	
	X-matrix	Y-matrix	X-matrix	Y-matrix	X-matrix	Y-matrix	**X-matrix**	**Y-matrix**
Fanyhessea vaginae	52%	16%	17%	16%	12%	15%	**81%**	**47%**
Prevotella bivia	53%	16%	18%	16%	9%	16%	**80%**	**48%**
Candida albicans dHp17	69%	13%	11%	15%	6%	16%	**86%**	**44%**
Candida glabrata	87%	19%	4%	13%	3%	13%	**94%**	**45%**

Table S2. Growth conditions and selected culture media for analyzed microorganisms

**Microorganism**	**Culture medium**	**Conditions**
Lactobacillus spp.	MRS	anaerobic conditions at 37 °C for 48h
Bifidobacterium spp.	MRS	anaerobic conditions at 37 °C for 48h
Streptococcus agalactiae	Chocolate agar	anaerobic conditions at 37 °C for 48h
Gardnerella vaginalis	MRS, RCM, TSA, Chocolate agar	anaerobic conditions at 37 °C for 48h
Atopobium vaginae,	MRS, RCM, TSA	anaerobic conditions at 37 °C for 48h
Prevotella bivia	MRS, TSA, Chocolate agar	anaerobic conditions at 37 °C for 48h
Finegoldia magna,	MRS, RCM, TSA, Chocolate agar	anaerobic conditions at 37 °C for 48h
Mobiluncus mulieris,	MRS, RCM, TSA, Chocolate agar	anaerobic conditions at 37 °C for 48h
Mobiluncus curtisii,	MRS, RCM, TSA, Chocolate agar	anaerobic conditions at 37 °C for 48h
Aerococcus tetradius,	MRS, RCM, TSA	anaerobic conditions at 37 °C for 48h
Anaerococcus christensenii	MRS, RCM, TSA	anaerobic conditions at 37 °C for 48h
Candida spp.	YPD medium	aerobic conditions at 37 °C for 24h
	MRS medium	aerobic conditions at 37 °C for 48h
Trichomonas vaginalis	L.Y.I. Entamoeba medium (ATCC-PRA-2154)	microaerophilic conditions at 35 °C for 48h

2. Figures

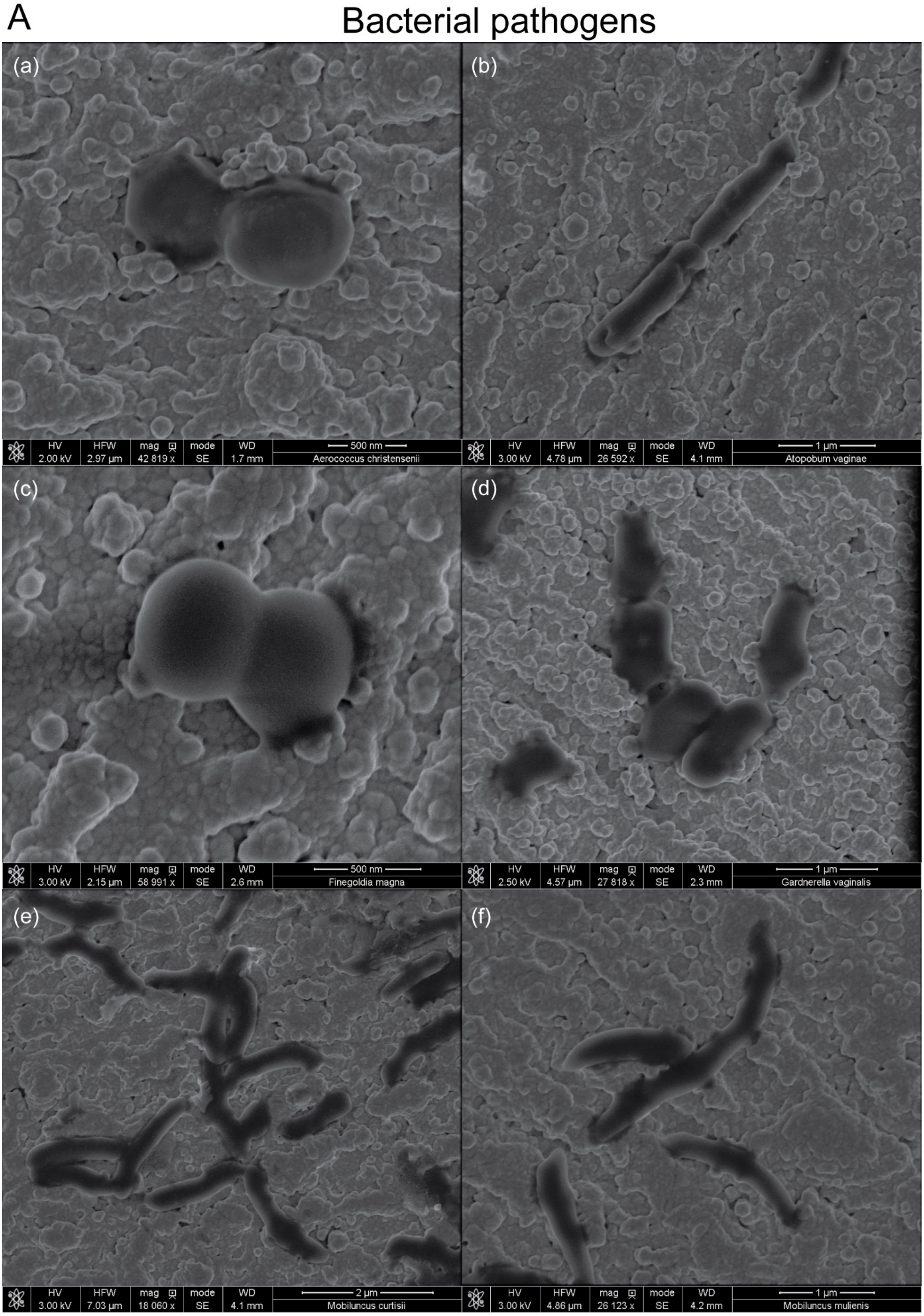

A
Bacterial pathogens
(a)
HV 2.00 kV HFW 2.97 µm mag 42 819 x mode SE WD 1.7 mm
500 nm
Aerococcus christensenii
(b)
HV 3.00 kV HFW 4.78 µm mag 26 592 x mode SE WD 4.1 mm
1 µm
Atopobum vaginae
(c)
HV 3.00 kV HFW 2.15 µm mag 58 991 x mode SE WD 2.6 mm
500 nm
Finegoldia magna
(d)
HV 2.50 kV HFW 4.57 µm mag 27 818 x mode SE WD 2.3 mm
1 µm
Gardnerella vaginalis
(e)
HV 3.00 kV HFW 7.03 µm mag 18 060 x mode SE WD 4.1 mm
2 µm
Mobiluncus curtisii
(f)
HV 3.00 kV HFW 4.86 µm mag 26 123 x mode SE WD 4.2 mm
1 µm
Mobiluncus mulienis

B *Lactobacillus* spp.

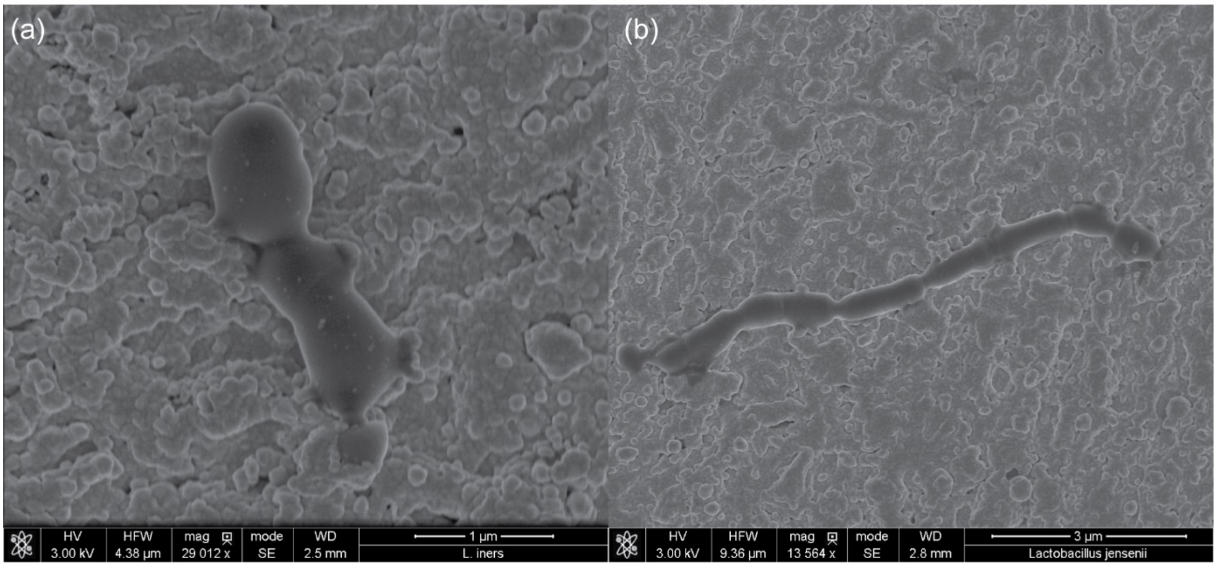

C *Bifidobacterium* spp.

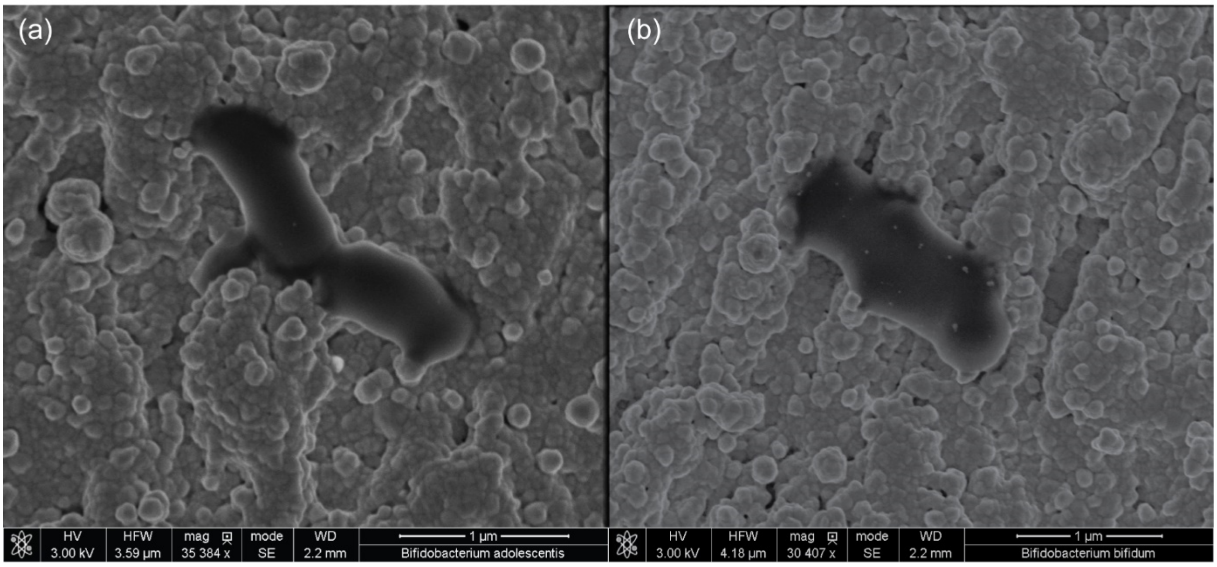

D Candida spp.

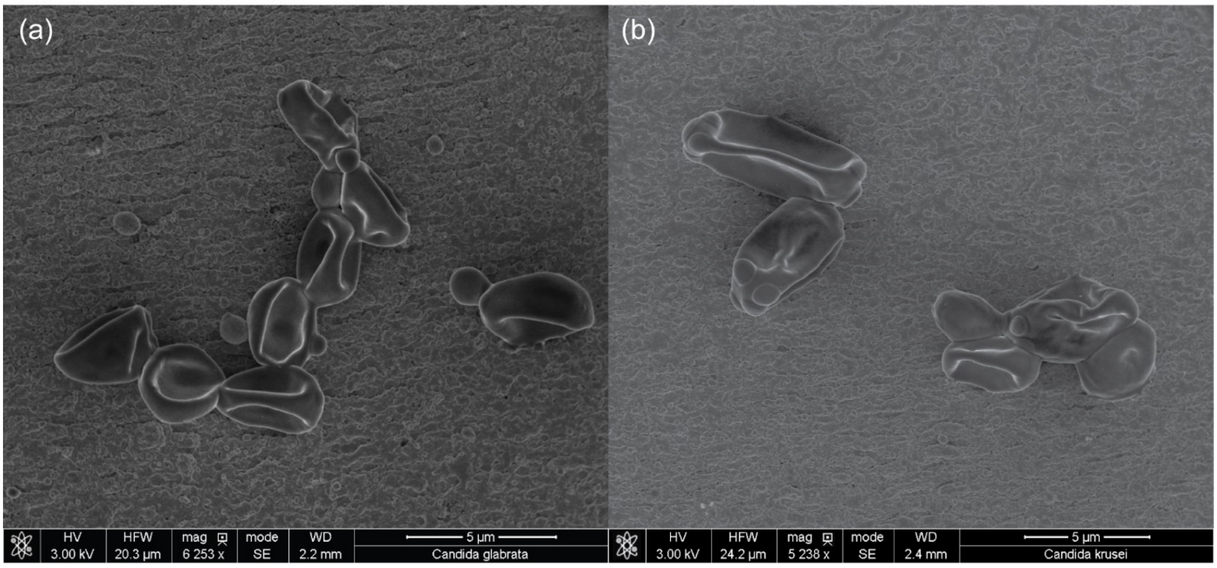

Figure S1. The SEM images of **A** bacterial pathogens a) *Aerococcus christensenii, b) Atopobium vaginae, c) Finegoldia magna,* d) *Gardnerella. vaginalis,* e) *Mobiluncus curtisii,* f) *Mobiluncus mulieris;* **B** *Lactobacillus spp.* a) *Lactobacillus iners,* b) *Lactobacillus jensenii;* **C** *Bifidobacetrum spp.* a) *Bifidobacterium adolescentis,* b) *Bifidobacterium bifidum;* **D** *Candida spp.* a) *Candida glabrata,* b) *Candida krusei* which are placed onto the Si/Ag SERS platforms.

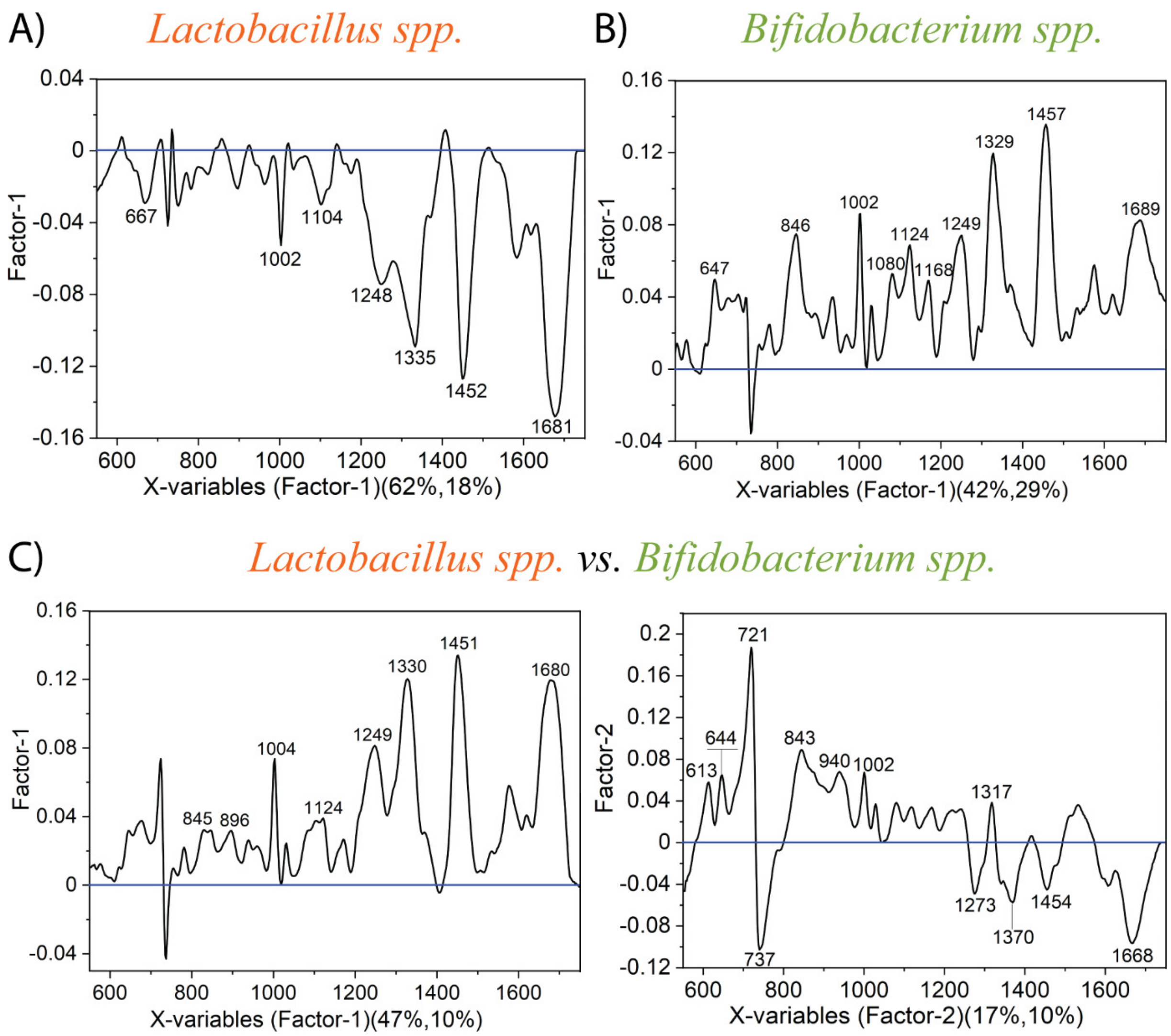

Figure S2. X-loadings plots for different associations (**A**) *Lactobacillus* spp., (Factor-1) (**B**) *Bifidobacterium* spp., (Factor-1) (**C**) *Lactobacillus* spp. and *Bifidobacterium* spp. together (Factor-1 and Factor-2)

a) *Pathogens cultured on RCM medium*

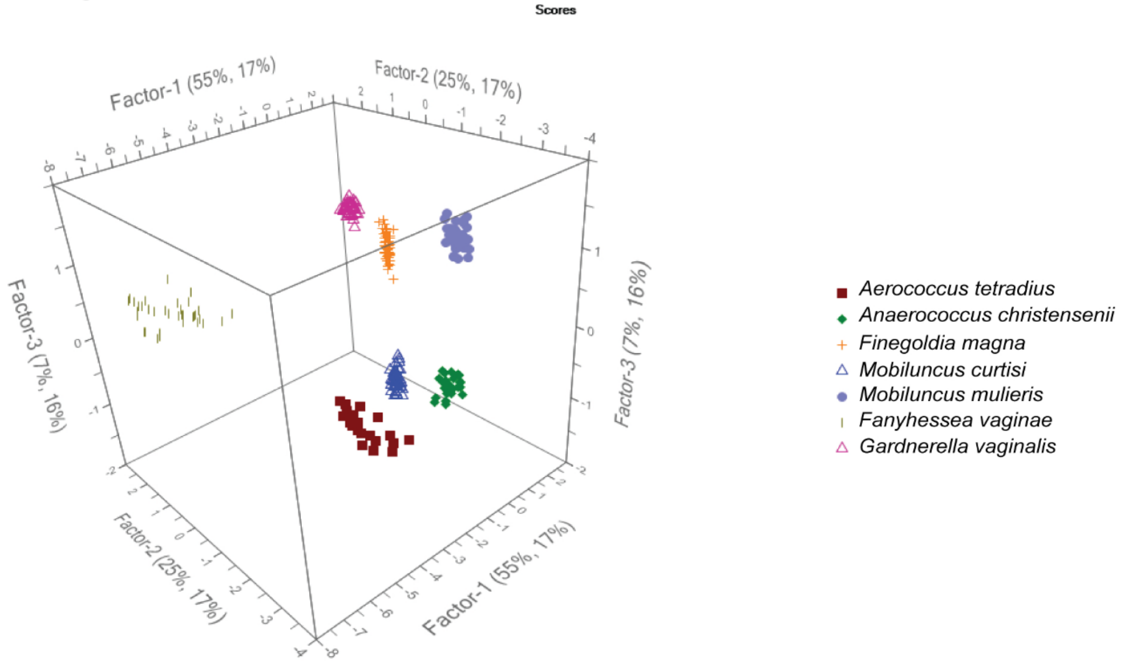

b) *Pathogens cultured on TSA medium*

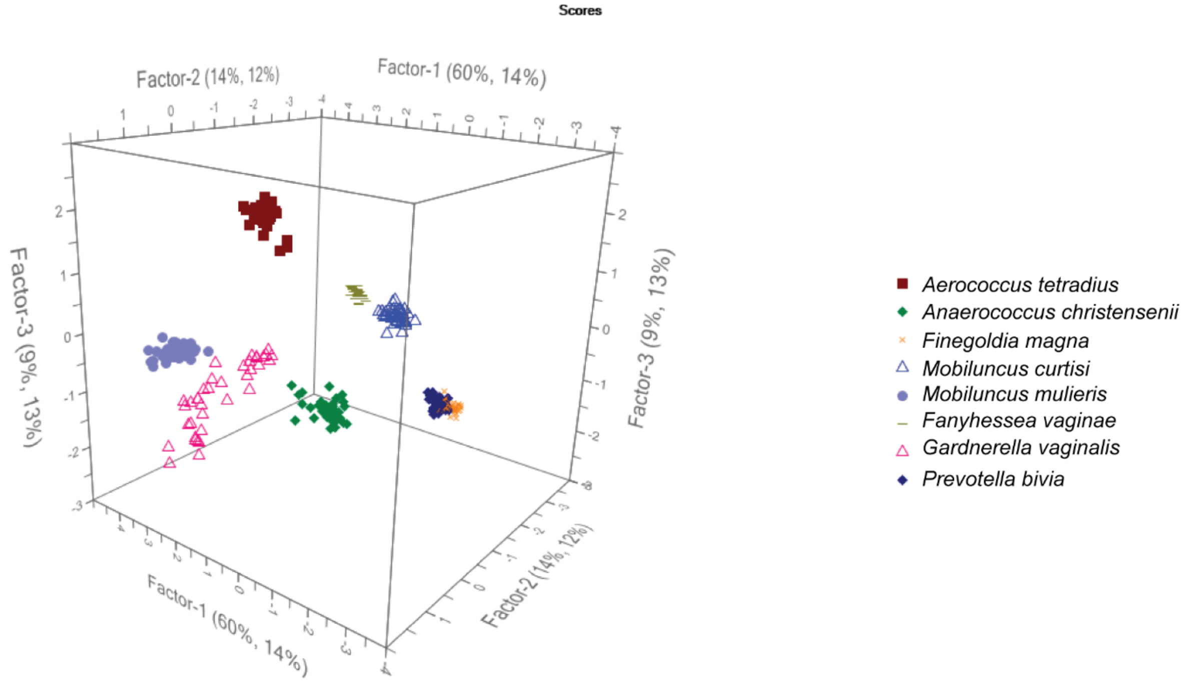

Figure S3. PLSR results in the form of score plots calculated for spectra of pathogenic bacteria that grown on (**a**) RCM and (**b**) TSA medium.

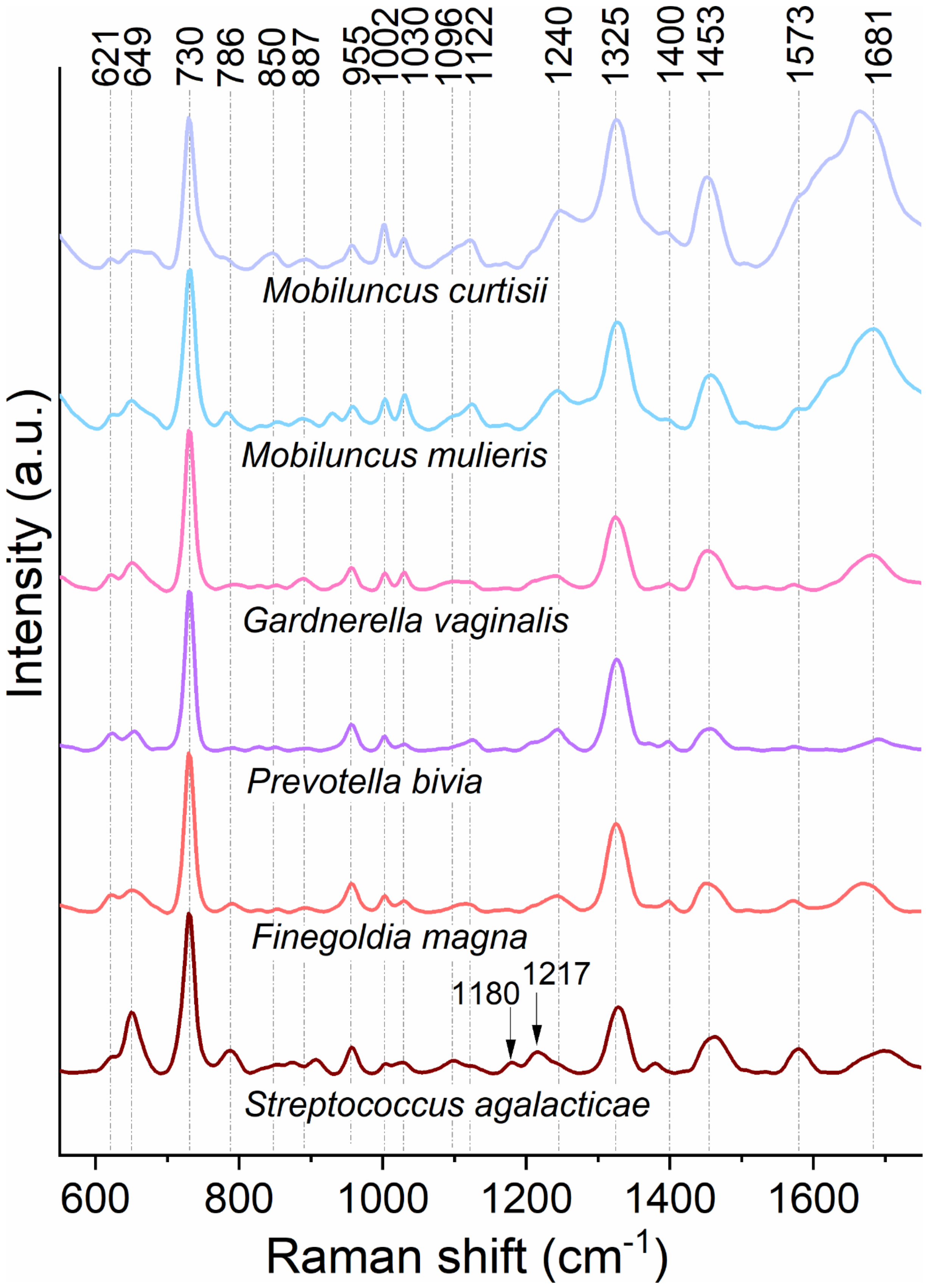

Figure S4. SERS spectra for pathogens that were cultured on chocolate medium

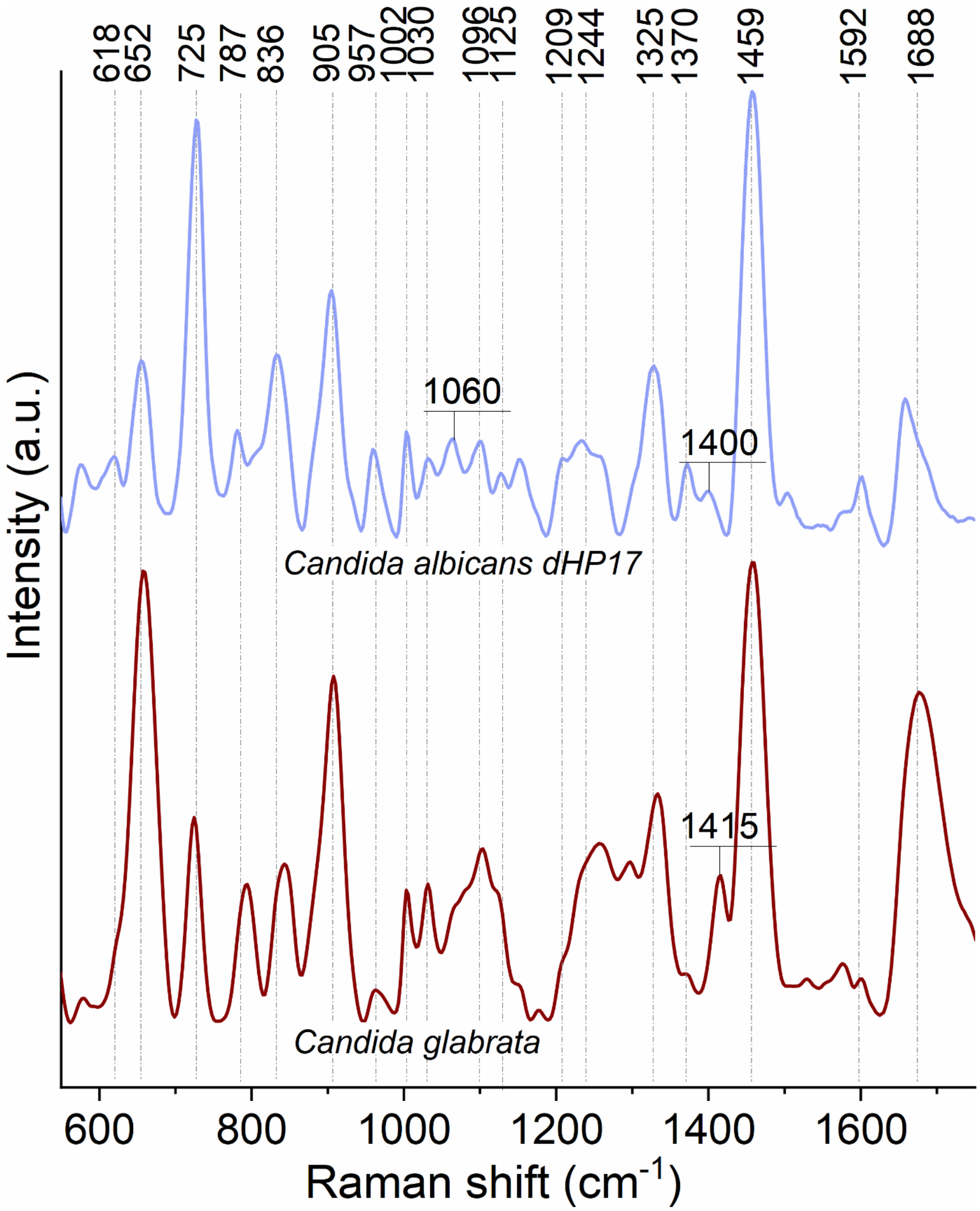

Figure S5. The SERS spectra for *C. glabrata*, *C. albicans dHp17* that were cultivated for 48h on MRS agar
